# Measuring early child development across low and middle-income countries: A systematic review

**DOI:** 10.1177/1476718X211020031

**Published:** 2021-06-14

**Authors:** Bernardita Munoz-Chereau, Lynn Ang, Julie Dockrell, Laura Outhwaite, Claire Heffernan

**Affiliations:** UCL Institute of Education, UK; UCL Institute of Education, UK; London International Development Centre (LIDC) London School of Hygiene and Tropical Medicine, UCL Institute of Education, UK

**Keywords:** child stunting, cognitive development, early childhood development, ECE setting, LMIC, home environment, systematic review

## Abstract

The Sustainable Development Goals mandate that by 2030, all children should have access to quality early child development opportunities, healthcare and pre-primary education. Yet validated measures of ECD in low and middle income countries (LMICs) are rare. To address this gap, a Systematic Review (SR) of measures available to profile the development of children between the ages of 0–5 years in LMICs was undertaken. Drawing on education, psychology and health databases, we identified reliable, valid or measures adapted for use in LMICs for either assessments of children’s development or their learning environments. The inclusion criteria were (1) peer reviewed papers published between January 2009 and May 2019; (2) assessment tools used to measure cognitive/language development or the early years or home environment in at least one LMIC; (3) report of the psychometric properties (validity and reliability) of the tool, and/or description of the cultural adaptability/translation process undertaken before applying it to a LMIC. Two hundred and forty-nine available records published in the last decade in peer-review journals and nine relevant systematic literature reviews were identified. Fifty-seven records were qualitatively synthesised based on their psychometric properties and cultural adaptation. Forty-three tools were reviewed utilising 12 criteria. Five elements of analysis present in Tables 2 and 3 (study, population tested, validity, reliability and cultural adaptability/translation) focused on the tools’ psychometric properties and previous application in LMICs. A further seven dimensions outlined in Tables 4 and 5 identified specific characteristics of the tools from target age, administration method, domains, battery, accessibility, language and country/institution. We suggest these 12 key considerations for the selection of measurement tools that are applicable to effectively assess ECD in LMICs.

## Introduction: Opportunities and challenges of measuring child development and learning environments across LMICs

Supporting, monitoring and measuring ECD outcomes is a global priority (McCoy et al., 2018a). In 2015, the United Nations adopted the 2030 agenda for the Sustainable Development Goals (SDGs) to end poverty and advance human development. The need to monitor and measure ECD underpins many of the goals. Identifying the kinds of environments that support young children’s learning are critical to address developmental challenges. One such challenge is childhood stunting.

Childhood stunting is the impaired growth and development that children experience as a result of poor nutrition, repeated infection and inadequate psychosocial stimulation. Stunting not only reduces linear growth but has major consequences for a child’s overall development, including poorer cognition and educational performance, with lifelong impacts on economic and social wellbeing (Williams and Suchdev, 2017). Stunting goes beyond the interaction between poor diets, nutrition, infectious disease and poor sanitation. Evidence suggests that interventions involving psychosocial stimulation provide much larger effect sizes for cognitive and language outcomes for children who are stunted, than interventions that focus on nutritional supplements alone (Aboud and Yousafzai, 2015). The data thus demonstrate the need to examine the wider sociocultural contexts in which children develop, highlighting the importance of capturing the child’s biodevelopmental niche, that is, the interaction between the child’s biological, physical and social environments (Super and Harkness, 2002).

Given the challenges raised by childhood stunting and the multifactorial drivers and impacts on development, interdisciplinarity is required to capture development. Crucially, achieving this objective requires reliable, valid and culturally sensitive assessments to profile both children’s development and their learning environments. Identifying the impact of different biodevelopmental niches also necessitates comparisons across countries and settings.

This SR aims to identify and evaluate potential measures for use as part of a large-scale interdisciplinary study UKRI GCRF Action Against Stunting Hub (2019–2024) working in India, Indonesia and Senegal. Key to the success of the study is providing comparative robust data about children’s development and learning contexts. Our ultimate aim is to provide a set of key considerations (outlined in Tables 2–5) which should inform decisions about which tool to use when carrying out studies in LMICs in general, and to inform specifically the education and cognition workstream of the Action Against Stunting Hub.

Reliable and valid measures provide the opportunity to track development, target needs, evaluate the efficacy of interventions and capture the impact of challenges to development both from within the child (Dockrell and Connelly, 2013) and the environment (Cabell et al., 2011). Without valid, reliable contextually sensitive tools, capturing the impacts of interventions and ECD at scale across different cultural contexts is likely to be misleading as neither baselines nor trajectories will be comparable. However, profiling early development raises substantial challenges. It is a period marked by significant growth in language, cognition, motor development and socioemotional behaviour, so markers of development are both quantitatively and qualitatively different across the period. This makes prediction over time challenging and often unreliable (Dockrell et al., 2015) particularly when some skills will be at floor at the earliest testing points. For example, 6-month old infants will have no expressive language while other indicators of development such as some motor skills, can reach ceiling effects relatively quickly. These developmental patterns indicate the importance of using concurrent measures of development to identify patterns of need and to use developmental trajectories only when there is a population-based comparison as a benchmark. The ways in which a child’s developmental competencies can be measured, also varies. Profiling domains of development may be based on either criterion referenced measures or normative data; as we shall see normative data are often lacking in LMICs. Measures may involve direct assessment of the child’s skills through the use of standardised tests or observations, or be collected by using a proxy, such as a parent or teacher reports. Direct assessments of children’s skills provide more robust and valid measures of development.

The challenges for drawing comparisons across populations varies by type of measure and response format. For example, norm-referenced tests, which are reliable and valid, provide information about where an individual lies in comparison to peers of the same age. Norm-referenced tests can focus on hypothetical constructs such as non-verbal ability or specific abilities such as naming vocabulary. The basic principle of norm-referenced tests is to define a continuum of performance from lowest to highest and the measure assigned to a particular individual locates his/her position on that continuum relative to the standardisation sample. Tests can only provide appropriate norms if they are used for the population for which they were intended. Norms from high-income (often USA, UK and Australia) countries will not be appropriate for LMICs samples where children experience very different social contexts, languages and have access to different educational opportunities. By corollary, norms that are standardised on monolingual children may not be appropriate for bilingual or multilingual children. Norms must also be current as they become out-dated by about three points a decade (Trahan et al., 2014).

To augment child-level data in the early years, it is also important to capture the child’s learning environment, profiling both the home and the early years settings (Fernald et al., 2009, 2017). Both environments have the potential to support and enhance ECD. The home learning environment includes the physical characteristics but also, importantly, the interactions which occur between the child and their families or primary caregivers within the home. These interactions offer both implicit and explicit learning opportunities for the child. The home environment is a key predictor of cognitive and socio-emotional development, and its effects are evident throughout formal education (Bradley and Caldwell, 1976; Olson et al., 1990). Home interactions, particularly maternal responsiveness, mediate the impact of social disadvantage on development (Evans et al., 2010; Foster et al., 2005). The impact of the home environment is complemented by the opportunities afforded by the early years environment.

The ‘quality’ of early years settings impacts on children’s development (Sylva et al., 2006). Assessments of quality typically consider both structural (e.g. child ratios, group size, caregiver’s qualifications and training) and process factors (e.g. caregiving practises, children’s experiences and caregiver–child interactions) that promote learning and development (World Health Organization, 2004). Whilst the nature of the environment varies across different types of settings, there is a strong relationship between structural and observed process characteristics. For example, as with the data from the home environment, process features such as caregivers’ warmth and responsiveness (Perlman et al., 2016), directly impact on positive children’s outcomes. Environments with high quality processes offer children rich opportunities to interact with adults, peers and materials (World Health Organization, 2004). Key factors for maintaining quality in preschool settings include child-adult pedagogical interactions, the curriculum, learning materials, teachers’ perceptions of learning and professional development opportunities (Mathers, 2021; Rao et al., 2019). Whether the same constructs generalise to LMICs is an important empirical question. Current understanding indicates that assessing learning environments need to consider the specific cultural context of what makes a positive learning environment (Raikes et al., 2019). There are therefore strong empirical and theoretical reasons to profile children’s development, identifying strengths and needs, as well as capturing the learning environment. Even in countries in the global North -where a wide range of assessment tools have been developed and standardised- there remain significant debates about which measures to use for which children at which point of development and in which settings.

While more than 80% of the global childhood population resides in LMICs, most ECD measures come from high income countries (Rao et al., 2019). Child Development Assessment tools (CDATs) in LMICs tend to follow one of four formats (Sabanathan et al., 2015):

1. a standard western CDAT with no adaptations;
2. a western CDAT translated (linguistic equivalence) and/or adapted for the local cultural environment (cultural equivalence);
3. an amalgamation of a number of translated and/or adapted items from several different western CDATs; or
4. a locally developed, culturally specific CDAT consisting of original items designed to be relevant to the population of interest.

Each of these approaches raises challenges for use and interpretation. Locally developed tools limit comparison across countries and settings, reducing our understanding of biodevelopmental niches. By contrast, measures designed and standardised in more affluent western settings with no appropriate adaptations will not be culturally appropriate. Norms are likely to be inaccurate and developmental criteria identified in criterion referenced assessments may not be culturally appropriate. There are thus a series of questions that need to be considered in any study aiming to profile the skills of children in LMICs (McCoy et al., 2018a, 2018b). In addition to the challenges with standardisation, measures which rely on self-completion by parents or professionals need to consider the literacy level of the respondents and the way in which items are interpreted within particular contexts. As McCoy et al. (2018b) argue ‘few valid and reliable tools exist for capturing ECD at scale across cultural contexts’ (p.58). What remains clear is that assessment tools developed in high-income countries (HICs) need to be modified before they are applied in LMICs. Cultural adaptation includes (a) establishing the appropriateness of target items, (b) translation/back translation of the measure and the underlying construct(s), (c) adaptation of the content and the procedure of administration, (d) piloting and iterative testing of the tool (Fernald et al., 2009). Without cultural adaptation, there is no guarantee that the same underlying abilities are being captured (Sabanathan et al., 2015). In sum, measuring ECD across LMICs poses significant challenges that need to be recognised when reporting child development profiles and profiling ECD environments.

Accordingly, the following research questions guided our work:

(1) What assessment tools have been used by peer-review published studies conducted in the last decade in LMICs to profile children aged 0–5 years old’s cognitive development and learning environment?
(2) What assessment tools have been recommended by relevant previous systematic reviews to measure children aged 0–5 years old’s cognitive development and learning environment?

## Methodology and methods

To answer RQ1 the SR aimed to identify reliable and valid tools which can be used in LMICs to profile children’s cognition and their learning environment. To answer RQ2, we included previous relevant systematic literature reviews (see ** in References). The methods entailed a systematic searching and screening of published literature using a set of inclusion and exclusion criteria:

### Selection criteria

The inclusion criteria were:

- peer reviewed papers published between January 2009 and May 2019
- assessment tools used to measure cognitive/language development or the early years or home environment used in at least one LMIC
- report of the psychometric properties (validity and reliability) of the tool, and/or description of the cultural adaptability/translation process undertaken before applying the tool to a LMIC.

We excluded studies that:

- Included assessments tools that were developed, standardised and used only on HIC
- Applied the tool to age groups different from our study
- Did not provide information about the tool’s psychometric properties (validity and reliability) and/or a description of the cultural adaptability/translation processes.

### Search terms

**Figure 1.**
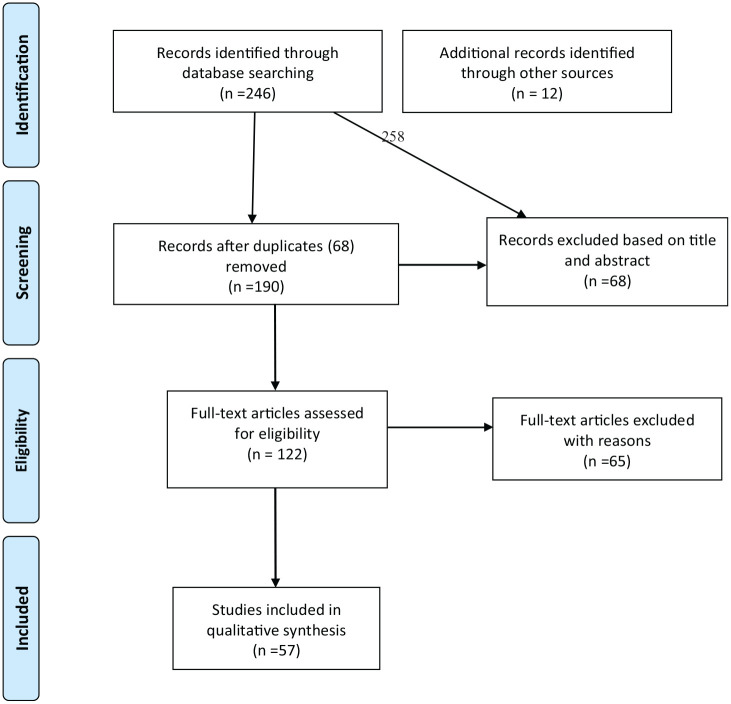
Preferred Reporting Items for Systematic Reviews and Meta-Analysis (PRISMA) guidelines flowchart for article selection (Moher et al., 2015).

Using the search terms provided in Table 1, 258 peer-review journal articles were retrieved through the authors’ university access system in relevant Education, Psychology and Health databases (ProQuest, PubMed, EconLit, PsychInfo, ERIC, Medline and Global Health). Two hundred and forty-six of these records were identified through database searching and 12 additional records were identified through hand search. Out of these, 68 duplicates where removed. From the 190 records screened, 68 were excluded based on their titles and abstracts following the inclusion and exclusion criteria descried above. After assessing 122 full-text articles, 65 were excluded as they did not meet the inclusion criteria. The remaining 57 full text articles were included in our qualitative synthesis (see * in the Reference). In order to ensure the accuracy and reproducibility of the review, the fourth author replicated the screening and data extraction stages. The raw proportion of agreement between both coders was very high (97%). After providing further evidence to justify the inclusion/exclusion criteria of the nine studies where there was disagreement (3%), one study previously excluded was included. Figure 1 shows the Preferred Reporting Items for Systematic Reviews and Meta-Analysis (PRISMA) guidelines flowchart for article selection (Moher et al., 2015).

**Table 1.** Search terms.

TERMS	AND	AND	AND
‘Assessment’, ‘questionnaire’, ‘checklist’, ‘tool’, ‘scale’, ‘measure’, ‘test’	‘Development’, ‘cognitive’, ‘cognitive development’, ‘cognition’	‘Child’, ‘infant’, ‘preschool’, ‘early childhood’, ‘early childhood education’, ‘early childhood education and care’	‘LMIC’, ‘Low-resourced setting’, ‘Global south’, ‘developing countries’, ‘low-income countries’, ‘low resource setting’
	OR		
	‘Learning environment’, ‘environment’		

### Data extraction and coding

Data from the 57 selected studies (43 reporting measures of child development, 14 environment) were entered into a spreadsheet. We extracted information from the studies using 25 criteria included in previous relevant SRs and agreed between the team: (a) Tool information (nine criteria); (b) Study information (four criteria), and (c) Tool application (12 criteria) (see Supplemental Appendix 1 for details). When information required for a full assessment of the feasibility of applying each tool was not provided, we imputed ‘not reported’ and interpreted it as an inconclusive area for future examination.

## Results: Qualitative synthesis

Forty-two selected studies included in the SR reported 34 tools assessing children’s development at age 0–5 years old in 35 LMICs. Most of the tools reported validity (*n* *=* 15), but this was variably described; some studies mentioned that the tool had ‘well established’, ‘satisfactory’ or ‘good’ validity without providing more details. Studies also varied in which type of validity was considered including concurrent, face, construct, content and convergent, without justifying these choices. When internal consistency was measured, Cronbach alpha varied between 0.23 (CREDI) and 0.95 (IDELA and CDSC). For most of the tools (*n* *=* 16), reliability (inter-rater and test-retest) was also reported. Results varied greatly from poor (Kappa: 00 for some CREDI items) to very good reliability (BSID-I, STBAPD and MacArthur-Bates Communicative Development Inventory 0.99). Indeed, differential reliability (per domain as opposed to general) was reported, with lowest coefficients for social–emotional, and highest for motor, cognitive and language domains. For most of the tools, (*n* *=* 15), cultural adaptation mentioned translation, backtranslation and adaptation of items to the new culture by the research team informed by local and international staff.

Nine environment tools were identified from the review. Ten studies reported five environment tools to measure the home environment at 0–5 years old in LMICs. These were applied in Bangladesh, Colombia, India, Indonesia, Mexico and Pakistan. Similarly to the developmental tools, there was marked heterogeneity in the way psychometric properties and cultural adaptation were reported. Again, there was variability in the type of validity reported including concurrent, face, construct, content and convergent, without justifying these choices. When internal consistency was measured, Cronbach alpha varied between adequate (Cronbach: 0.71 for FCI) to moderate (0.46 HSQ). Some studies simply referred back to previous research stating that the tool had good test-retest reliability or high inter-rater reliability without providing specific details. Cultural adaptation tended to mention that the instrument has been used worldwide previously, but with limited information regarding the process undertaken. Only the HOME included detailed information regarding its cultural adaptation.

From the five studies reporting four environmental tools to measure ECE settings, these were applied in four LMICs: China, Indonesia, Tanzania and South-Africa. Only the Chinese Early Childhood Environment Rating Scale (CECERS) mentioned good content, concurrent and criterion-related validity and the Early Childhood Environment Rating Scale-Revised (ECERS-R) referred to demonstrated predictive validity. The reliability of the three tools was reported to be good, ranging from 0.95 for ITERS-R to 0.97 for ECERS-R. Cultural adaptation tended to mention that the instrument has been used worldwide previously, but with limited detailed information regarding the process undertaken, except for Measuring Early Learning Quality and Outcomes (MELQO) (MELE module) where the process undergone was outlined.

Focusing on the 34 tools to assess children’s development at age 0–5 years old in LMICs, their target age was (1) 18–24 months (*n* *=* 9); (2) 25–60 months (*n* *=* 21) and 0–60 months (*n* *=* 4). Focusing on the administration method, the majority of assessments were direct assessments of the child (23) and 11 were completed by caregivers. Developmental domains included language, cognition, motor skills and social-emotional development. However, the operationalisation of the domains varied by test and developmental phase. Overall, language (*n* *=* 22) and cognition (*n* *=* 17) were assessed in the majority of measures while motor skills (*n* *=* 16) and socio-emotional development (*n* *=* 14) were less common. More than a third of the studies did not include information about accessibility (*n* *=* 11). From those that did, 14 required payment and nine were free to use. The tools were primarily produced in English (*n* *=* 24), with five tools developed in local languages, such as French, Kigirima and Chinese. Five tools did not report the language of use. Most of the tools were developed in USA (*n* *=* 13), while others were globally developed by international organisations such as World Bank, UNICEF and UNESCO (*n* *=* 6). A few were developed in the UK (*n* *=* 5) and countries such as India, Malawi, Kenya, Hong Kong and South Africa.

Focusing on the ten studies reporting five environment tools to measure the home environment at 0–5 years old in LMICs, all were suitable for 0–60 months (*n* *=* 5). Regarding the administration method, the majority of the tools were completed by caregivers (*n* *=* 4), and one was a direct assessment. Three tools focused on cognitive and socioemotional caregiving with no information provided for the remaining two. Information about accessibility was often not provided (*n =* 3), while two required payment. The dominant language of the tools was English (*n =* 4), with one tool with missing information. Most of the tools were developed by international organisations such as UNICEF (*n =* 3), with the remaining two, developed in USA and India, respectively.

Five studies reported four tools to measure the early learning environments at age 0–5 in LMICs. Regarding the target age, one was suitable for 0–60 months (*n =* 1), and three, for 25–60 months (*n =* 3). Three of the tools were direct assessments (*n =* 3), one parent reported, and one was not reported. The environment tools also vary in terms of the domains assessed. All assessed the space and physical setting as well as the quality of interactions, curriculum planning and implementation and personnel (*n =* 4), but varied in terms of the other included dimensions, such as personal care routines (*n =* 2), inclusiveness (*n =* 1) and play (*n =* 1). Scales which examined the environment and physical setting were more common in the ECE settings measures than the home environment, whereas the key feature included in every tool was the quality of interaction with the child. Regarding accessibility, two studies did not include this information, one required payment and one was free to use. Two tools were in English (*n =* 2), one in Chinese, and one was not reported. Two of the tools were global (*n =* 1), one developed in USA (*n =* 1), and one was Chinese (*n =* 1).

## Discussion: 12 considerations for selecting suitable measurements to effectively assess ECD in LMICs

The SR was undertaken to identify tools available to profile the development of children and their learning environments between the ages of 0–5 years. It reviews forty-three tools (34 focusing on child development and 9 on the environment) that have been used previously to assess early development in LMICs. The ongoing debate about which measure to use for which children in which setting remains a pressing one. This is of particular importance for childhood stunting and, as such, for the UKRI GCRF Action Against Stunting Hub.

Drawing on the synthesis of 57 records included in this SR, we compared the tools’ application identifying five critical markers (the study, population tested, validity, reliability and cultural adaptability/translation) outlined in Tables 2 and 3. Focusing on the psychometric properties, studies varied greatly in the way validity and reliability were reported, ranging from no reporting to a variation in the way these characteristics were addressed. Studies also varied in which type of validity was considered including concurrent, face, construct, content and convergent, without justifying these choices. Without valid and reliable tools, measuring the impacts of developmental challenges, such as stunting, interventions and ECD across different cultural contexts will not yield equivalent conclusions making it harder to identify barriers, drivers of development, and effective interventions. Here we used as a benchmark validity >.7, but a close attention of how reliability is reported is important. Regarding the cultural and contextual appropriateness/potential to adapt, and in line with Sabanathan et al. (2015), we found that researchers typically translated tools from HIC (linguistic equivalence) with a minority adapting them in a systematic way for the local cultural environment (cultural equivalence). In some cases, an amalgamation of a number of translated and/or adapted items from several different HIC tools were used but the validity of this approach was rarely examined. Researchers need to actively engage in developing robust measures which include cultural adaptations and translation/back translation. These procedures and any changes should be reported for the measures.

**Table 2.** Development assessment tools by name, study, population tested, validity, reliability and cultural adaptability/translation.

N	Tools	Study	Population and sample	Validity	Reliability	Cultural adaptability/translation
1	Caregiver-reported early child development (CREDI)	McCoy et al. (2017)	Peri-urban and rural Tanzania 2481 children	Internal consistency: 0.68–0.90	Inter-rater reliability: 0.00–0.40	Back/translated to/from Swahili by bilingual Tanzanian and American staff. Discrepancies resolved on consensus of a committee formed by CREDI, local staff and bilingual Tanzanian community members
		McCoy et al. (2018a)	LMICS including India 8022 children	Criterion validity: 0.34–0.92 Age-normalised correlations: 0.23–0.47	Test retest reliability: 0.60	Back/translated all CREDI items and materials into local language(s). Teams referenced to CREDI item descriptions and used colloquial language. Local teams made minor adaptations to the item examples
		Boggs et al. (2019)	LMICs including India (SLR)	Rated excellent. Exact values not reported	Rated highly. Exact values not reported	Not reported in SLR
2	Developmental milestones checklist (DMC)	Prado et al. (2014)	Burkina Faso 1123 children	Internal consistency: 0.70–0.88 Sensitive to age and group differences	Inter-rater reliability: 0.85–0.93 Test retest reliability: 0.77–0.96	Flexible administration procedure to allow observations and caregiver interviews. Modified form and manual were translated into French by two bilingual French and English speakers
3	Developmental milestones checklist (DMC II)	Larson et al. (2017)	Rural India 4360 children	Internal consistency: 0.67–0.94	Inter-rater reliability: 0.96	Back/translated into English and Hindi. Full survey piloted on children from Bihar and adapted. The order of questions in the personal-social subscale was adapted
4	Intergrowth-21st (inter-NDA)	Fernandes et al. (2014)	Brazil, India, Italy, Kenya and UK 4607 children	Not reported	Inter-rater reliability: 0.70 Test-retest reliability: 0.79	Site team compiled, discussed and solved item-based culture-specific issues. Amendment of phrases to more culturally appropriate conceptual equivalents. For maternally reported items on attention and emotional reactivity, local language versions (Brazilian Portuguese, Hindi, Italian, Kiswahili and Marathi) from validated CBCL translations were made available to site staff
		Semrud-Clikeman et al. (2017)	LMICS including India and Indonesia (SLR)	Not reported in SLR	Inter-rater reliability: 95% Test retest reliability: 95%	Not reported in SLR
5	Bayley MDI	Fernald et al. (2017)	LMICs including Indonesia (SLR)	Not reported	Not reported	Not reported
6	Bayley scales of infant development I (BSID-I)	Luo et al. (2015)	Rural China 1808 children	Not reported	Inter-rater reliability: 0.99 Test retest reliability: 0.82–0.88 Parallel forms reliability: 0.85–0.87	The test was adapted to Chinese language and environment in 1992 in an urban Chinese sample
7	Bayley scales of infant development II (BSID-II)	Carlo et al. (2013)	India, Pakistan and Zambia 371 children	Pilot-testing at each site to verify validity in the local context. Exact values not reported	Not reported	Few items were slightly modified to make it more culturally appropriate (i.e. image of a sandal instead of a shoe)
		Fernald et al. (2009)	LMICs including Indonesia (SLR)	Not reported in SLR	Not reported in SLR	Not reported in SLR
		Wallander et al. (2014)	India, Pakistan and Zambia 145 children	Pilot-testing at each site to verify validity in the local context. Exact values not reported	Not reported	Few items were slightly modified to make it more culturally appropriate (i.e. image of a sandal instead of a shoe)
8	Bayley scales of infant development III (BSID-III)	Bhopal et al. (2019)	Rural India 1726 children	Not reported	Inter-rater reliability: 97%	Systematic cultural adaptation process: (1) translation into Hindi independently by two trained research associates, (2) comparing translations and assessing technical equivalences, then producing final translations by consensus, (3) field research with project staff and mothers of young children to test understanding of translations and to improve them, (4) finalisation of tool for pretesting and (5) pretesting in the community
		Luo et al. (2019)	China 448 caregiver–child dyad	Not reported	Not reported	No adaptations made
		Wallander et al. (2010)	India, Pakistan and Zambia 431 children	Not reported	Not reported	Extensive testing and cultural adaptation through training on three occasions and ongoing evaluators’ monitoring
		Kirsten et al. (2018)	South Africa 1143 children	Not reported	Not reported	Translated measures into Afrikaans and isiXhosa using standard forwards and backwards-translation method
		Nores et al. (2019)	Colombia 459 children	Not reported	Not reported	Translation provided under a licence by the publisher (Pearson) done for another study on a similar population in Colombia with a test-retest reliability 0.95–0.98 (Attanasio et al., 2014)
		Semrud-Clikeman et al. (2017)	LMICs including Indonesia (SLR)	Criterion validity with McCarthy scales and WPPSI: 0.73–0.79 Internal consistency: 0.89	Test retest reliability: 0.83	Not reported in SLR
9	Kilifi developmental checklist (KDC)	Sabanathan et al. (2015)	LMICs including Senegal (SLR)	Rated excellent. Exact values not reported	Rated excellent. Exact values not reported	Items chosen based on ease of observing item success, ability to differentiate within study population, and ease to describe in local language
10	Lucknow development screen for Indian children	Fischer et al. (2014)	LMICs including India (SLR)	Sensitivity: 95.9% Specificity: 73.1% Criterion validity with developmental assessment scale for Indian infants, and the vineland social maturity scale; exact values not reported	Not reported in SLR	Not reported in SLR
11	WHO indicators of infant and young child development (IYCD)	Lancaster et al. (2018)	LMICs including India and Indonesia 21,083 children	Rated excellent. Exact values not reported	Not reported	Not reported
12	East Asia-pacific early child development scales (EAP-ECDS)	Rao et al. (2019)	Cambodia, China, Mongolia and Vanuatu 4712 ethnic majority children	Rated excellent. Exact values not reported	Inter-rater reliability: 85%	Culturally developed
		Sun et al. (2018)	Cambodia, China, Mongolia, Papua New Guinea, Timor-Leste and Vanuatu 7583 children	Rated excellent. Exact values not reported	Inter-rater reliability: 85%	Culturally developed
13	International development and early learning assessment (IDELA)	Pisani et al. (2018)	LMICs including India and Indonesia	Convergent validity: 0.33–0.61 Internal consistency: 0.66–0.95	Inter-rater reliability: 0.79–0.97	Not applicable
		Yousafzai et al. (2018)	Rural Pakistan 340 children	Not reported	Inter-observer reliability: 0.99	Questionnaires and child assessments administered in Sindhi. Language and sociocultural adaptation protocols ensured original items conceptual integrity in adaptation
		Halpin et al. (2019)	Afghanistan, Bolivia, Ethiopia, Uganda and Vietnam 4970 children	Measurement invariance analysis revealed that most items do not provide a basis for comparing children’s development over the five countries	Not reported	Translated into Vietnamese, Luganda, Oromiffa, Spanish, Usnek and Dari
		Wolf et al. (2018)	108 public schools and 132 private schools, Ghana	Internal consistency: 0.69–0.83	Inter-rater reliability: 71.1%	The current protocol was piloted and adapted to the Ghanaian context. Specific details not included
14	Early childhood development index (UNICEF)	Urke et al. (2018)	Honduras 2729 children	Internal consistency: 0.41	Not reported	Not reported
15	Child developmental scale of China (CDSC)	Li et al. (2019)	9 Chinese provinces 2111 children	Internal consistency: 0.71–0.95 Criterion validity with Stanford-Binet intelligence scale: 0.60	Test retest reliability: 0.89	Tool developed in China
16	Hong Kong early child development scale (HKECDS)	Rao et al. (2013)	Hong Kong Central district and Tin Shui Wai 240 Chinese children	Internal consistency: 0.61–0.95	Inter-rater reliability: 90%	Characters used instead of letters in the language scale. Items developed in English and translated into bilingual Cantonese research assistants. Accuracy of translation evaluated by members of the research team who were very proficient in both languages
17	Early development instrument (EDI)	Brinkman et al. (2015)	Indonesia Sample size varies	Not reported	Not reported	Response options changed from five-point Likert scale to binary responses. Omitted questions that asked for the subjective evaluation of a child’s abilities. Wording of 11 questions altered to make their meaning clearer in the Indonesian language
18	Wechsler intelligence scales for children (WISC)	Fernald et al. (2017)	LMICs including India (SLR)	Not reported in SLR	Not reported in SLR	Not reported in SLR
		Semrud-Clikeman et al. (2017)	LMICS including India and Indonesia (SLR)	Not reported in SLR	Not reported in SLR	Not reported in SLR
19	Wechsler preschool and primary scale of intelligence-III (WPPSI-III)	Tarullo et al. (2017)	Rural Pakistan 105 children	Internal consistency: 0.69–0.88	Not reported	Cultural adaptation reported elsewhere (Rasheed et al., 2016)
		Semrud-Clikeman et al. (2017)	LMICs including India and Indonesia (SLR)	Not reported in SLR	Not reported in SLR	Not reported in SLR
		Jeong et al. (2018)	Rural Pakistan 1302 children	Internal consistency: 0.74	Not reported	Cultural adaptation reported elsewhere (Rasheed et al., 2018)
20	Measuring early learning quality and outcomes (MELQO) (MELE module)	Raikes et al. (2019)	Tanzania 684 children	Internal consistency: 0.32–0.91	Rated highly. Exact values not reported	Adaptation of direct assessment and the teacher report items previously used in LMICs
21	Peabody picture vocabulary test (PPVT)	Tomlinson et al. (2016)	South Africa 644 mother-child dyads	Not reported	Not reported	Cultural adaptation reported elsewhere (Pakendorf and Alant, 1996)
22	MacArthur-Bates communicative development inventory	Hamadani et al. (2010)	Rural Bangladesh 801 children	Not reported	test-retest reliability: 0.67–0.99	The inventory was developed after extensive piloting with mothers of young children and in consultation with experts
23	McCarthy scales for children’s abilities general cognitive index (MSCA)	Knauer et al. (2019)	Poor, rural Mexico 603 children	Not reported	Not reported	The MSCA were translated and adapted for use in Mexico by researchers at Instituto Nacional de Perinatología in Mexico City
24	British ability scales (BAS)	Fernald et al. (2009)	LMICs including India (SLR)	Not reported in SLR	Not reported in SLR	Not reported in SLR
25	Standford binet	Fernald et al. (2009)	LMICs including India (SLR)	Not reported in SLR	Not reported in SLR	Not reported in SLR
26	Kaufman assessment battery for children (KABC)	Fernald et al. (2017)	LMICs including Senegal (SLR)	Not reported in SLR	Not reported in SLR	Not reported in SLR
27	Kaufman assessment battery for children – second edition (KABC-II)	Semrud-Clikeman et al. (2017)	LMICs including Senegal (SLR)	Not reported in SLR	Internal reliability = 0.70–0.96	Not reported in SLR
28	Baroda development screening test (BDST)	Fischer et al. (2014)	LMICs including India (SLR)	Specificity: 65% Sensitivity: 95%	Not reported in SLR	Not reported in SLR
29	Developmental Neuropsychological Assessment (NEPSY-II)	Semrud-Clikeman et al. (2017)	LMICs including Indonesia (SLR).	Internal consistency = 0.88–0.94	Reliability = 0.28–0.88	Not reported in SLR
30	Malawi developmental assessment tool (MDAT)	Gladstone et al. (2010)	Malawi 1426 children	Rated satisfactory. Exact values not reported	Inter-rater reliability: >0.4	Consistency and clarity of items was ensured by back translating the tool with help of language expert from the University of Malawi. Items illustrated by a Malawian artist
		Boggs et al. (2019)	LMICs including India (SLR)	Rated excellent. Exact values not reported	Rated highly. Exact values not reported	Not applicable
		van den Heuvel et al. (2017)	Malawi 150 children	Internal consistency: 0.76–0.84	Inter-rater reliability: 0.98	Translation not needed
31	Ages and stages questionnaire 3rd ed. (ASQ-3)	Bernal et al. (2019)	Colombia 2767 children	Internal consistency: 0.87	Not reported	The ASQ has been used for early development assessments in LMICs (Rubio-Codina et al., 2015, in Bernal et al., 2019: 422)
		Nair et al. (2017)	Rural Bangladesh 1018 children	Not reported	Inter-observer reliability: 0.93–1.0	Version translated into Bangla and adapted to the local context by a team leading expert in child development. Adaptations made reflected relevant examples for the Bangladeshi context during the assessment while preserving the original questions from the ASQ
		Scherer et al. (2017)	Pakistan 868 mother-child dyads	Not reported	Not reported	Not reported
		Knauer et al. (2018a)	Poor, rural Mexico 1893 children	Not reported	Not reported	The EASQ was adapted for use in Mexico by researchers at the Instituto Nacional de Perinatología, Mexico City
		Kvestad et al. (2015)	North India 422 children	Rated excellent. Exact values not reported	Rated highly. Exact values not reported	Forms translated to Hindi following official recommendations. Items not appropriate for the cultural setting were identified and adjusted. Exact items adjusted not identified
		Knauer et al. (2018b)	Poor, rural Mexico 603 children	Not reported	Not reported	Not reported
32	ICMR (Indian Council Medical Research) psychosocial developmental screening test	Fernald et al. (2009)	India (SLR)	Not reported in SLR	Not reported in SLR	Not reported in SLR
		Semrud-Clikeman et al. (2017)	LMICs including Indonesia, and India (SLR)	Not reported in SLR	Not reported in SLR	Not reported in SLR
33	Screening test battery assessment psychosocial development (STBAPD)	Fischer et al. (2014)	LMICS including India (SLR)	Not reported in SLR	Inter-tester reliability: 95%–98% Retest reliability: 95%–99%	Not reported in SLR. Developed by Vazir et al. (1994)
34	TDSC (Trivandrum developmental screening chart)	Chattopadhyay et al. (2015)	Rural India 427 children	TDSC based on Bailey Developmental Screening Tool validated in India	Not reported	Developed in India

**Table 3.** Environment (Home and ECE setting) assessment tools by name, study, population tested, validity, reliability and cultural adaptability/translation.

N	Tools	Study	Population and sample	Validity	Reliability	Cultural adaptability/translation
Home environment						
1	Infant/toddler (IT) HOME, early childhood (EC)	Nores et al. (2019)	Colombia, 819 children	Not reported	The inter-rater reliability was above 0.9 on the full scale	The assessment instruments chosen have been used extensively in evaluations of early care and education including studies in developing countries (Fernald et al., 2017)
		Hamadani et al. (2010)	Rural Bangladesh, 801 children	Validated instrument across countries	The intraclass correlation for each of the four interviewers with the trainer ranged from *r* = 0.94–0.99 (*n* = 20)	Although the HOME is a good measure of the home environment, the scale is not suitable for use in large-scale population surveys. The HOME takes 45–60 minutes to administer and requires skilled, well-trained interviewers and considerable adaptation when used in developing countries. Moreover, the HOME involves observations, which are more difficult to standardise for use in large population surveys
		Knauer et al. (2018)	Poor, rural Mexico, 1893 children	The items that comprise the instrument were selected based on empirical evidence, and then validated through testing. The instrument has been well validated in the U.S. and used worldwide, including in several Latin American countries	The internal consistency of the scale was satisfactory (Cronbach = 0.8227)	Not reported
		Knauer et al. (2018)	Poor, rural Mexico, 603 children	The HOME Inventory has been well validated and used worldwide	The internal consistency of the scale was satisfactory (Cronbach = 0.8227)	Not reported
		Scherer et al. (2019)	Pakistan, 869 dyads (mother and child)	This tool, designed to assess home environment and stimulation quality, has been used frequently in LMIC	Not reported	Not reported
2	Family Care Indicator (FCI) questionnaire	Knauer et al. (2018)	Poor, rural Mexico, 603 children	Has been well validated, Cronbach = 0.7100	Not reported	Has been used worldwide
		Hamadani et al. (2010)	Bangladesh, 801 children	Determining the validity of the FCIs across cultures requires studies in a number of cultural settings and is beyond the scope of this study	To assess short-term test-retest reliability, the FCI questionnaire was repeated 7–14 days later among 40 mothers. The items that were observed (‘household books’, ‘magazines’, ‘varieties’ and ‘sources’ of play materials) were highly reliable (intraclass correlations *r* > 0.85, *p* < 0.001) whereas ‘play activities’ was only moderately reliable (*r* = 0.64, *p* < 0.001)	Not reported
3	Home Screening Questionnaire (HSQ)	Nair et al. (2009)	India	The likelihood ratio (LR) for positive test was .46 (95% CI, 3.3 to 6.9)	the sensitivity was 83%, specificity 82%, positive predictive value 83.3%, negative predictive value 81.6%, and accuracy 82.5%	Not reported
4	Multiple Indicator Cluster Survey (MICS) two questionnaires: Household Questionnaire. Questionnaire for Individual Women	McCoy et al. (2018)	Nationally representative data collected in 58 LMICs	The activities have been previously found to show acceptable predictive validity when relating to child outcomes	The activities have been previously found to show acceptable short-term reliability	All survey questions are translated and back translated into local languages by in-country teams
5	Multiple Indicator Cluster Survey (MICS 3) three questionnaires: Household Questionnaire. Questionnaire for Individual Women, and a Questionnaire for Children Under Five	Bornstein et al. (2012)	28 developing countries, 127,000 families with under-5 years children	Not reported	Kuder-Richardson 20 reliabilities were satisfactory (DeVellis, 2003) at 0.68 for the cognitive caregiving scale	MICS3 covers a large array of topics and its significant flexibility allow countries to adapt the survey to their particular situations and needs but keeps comparability through standardised questions and administration
ECE centre						
1	Infant/toddler environment rating scale-revised (ITERS-R); early childhood environment rating scale-revised (ECERS-R)	Brinkman et al. (2016)	Indonesia, 310 total villages sampled, with 4–16 students observed within each village	Many studies have demonstrated its predictive validity (Burchinal et al., 2008; Montes et al., 2005; Peisner-Feinberg et al., 2001)	Good test-retest reliability, high inter-rater reliability (Clifford et al., 2010)	Not reported
	Infant/toddler environment rating scale-revised (ITERS-R); early childhood environment rating scale-revised (ECERS-R)	Biersteker et al. (2016)	South Africa, 242 centres	Not reported	Good reliability ITERS-R subscales ranging from 0.68 to 0.88, with an overall scale of 0.95. Sound overall good reliability was observed for the two instruments ranging from 0.68 to 0.92, with an overall scale reliability of 0.95 for ITERS-R and 0.97 for ECERS-R	For the ITERS-Rand the ECERS-R, there was 70% agreement on all items rated and 83% within one point of the 7-point scale. Assessors who did not fall within this range were not retained
2	Child Activities (CA) system	Montie et al (2006), in Fernald et al. (2009)	Indonesia	Not reported	Not reported	Not reported
3	Chinese Early Childhood Environment Rating Scale (CECERS)	Li et al. (2019)	8 Chinese provinces, 2110 children	Good content validity, concurrent validity and criterion-related validity	Good reliability within each sub-scale and the total score. Internal consistency (Cronbach’s alpha) ranged from 0.83 to 0.93 for the subscales and was 0.96 for the total scale	Not reported
4	Measuring early learning quality & outcomes (MELQO) (MELE module)	Raikes et al. (2010)	Tanzania, 684 children	Not reported	Enumerators were evaluated several times with accuracy checks and were judged to reach reliability standards based on participation in in-person training and two checks on reliability conducted at the end of the training period	Adaptation of direct assessment and the teacher report items previously used in LEMICs; items commonly-used in intelligence and school-readiness instruments (i.e. IDELA) (Wolf et al., 2017), head shoulders knees toes task (McClelland et al., 2014), and the Canadian National Longitudinal Study of Children and Youth and the EDI (Janus and Offord, 2007)

We also compared 43 tools (34 for assessing children’s development, 5 to measure the home environment and 4 to measure ECE settings) on key markers outlined in Tables 4 and 5. We suggest that these seven markers (target age, administration method, domains, battery, accessibility, language and country/Institution) are also critical to address the implementation challenges that practitioners and researchers face when choosing tools. Crucially, information on the tool’s accessibility (including licences, training and other operational aspects) are required in order to successfully apply the tool to a new context. Moreover, measures which require high levels of professional training will be challenging in contexts where psychologists, speech and language therapists or occupational therapist are not commonplace.

**Table 4.** Development assessment tools by name, target age, administration method, domains, battery, accessibility, language and country/Institution.

N	Tool’s name	Tool’s target age	Administration method	Domains	Battery	Accessibility	Language of the tool	Country/Institution
1	Caregiver Reported Early Childhood Development Instruments (CREDI)	1	Caregiver report	Motor, cognitive, language, social-emotional and mental health development	No	Free to use	English	USA
2	Developmental Milestones Checklist (DMC)	1	Caregiver report	Motor, language and personal-social development	No	Free to use	English	Kenya
3	Developmental Milestones Checklist II (DMC II)	2	Caregiver report	Social/emotional; cognitive (learning, thinking, problem-solving); language/communication; movement/physical development	Yes	Free to use	French	USA
4	Intergrowth-21st (inter-NDA)	1	Direct assessment	Cognition, language skills, behaviour, motor skills and attention	Yes	Free to use	English	UK
5	Bayley MDI	2	Direct assessment	Achievement, simultaneous processing and sequential processing	Not reported	Not reported	Not reported	World Bank
6	Bayley scales of infant development I (BSID-I)	1	Direct assessment	Motor, cognitive and language scales	Yes	Payment required	English	USA
7	Bayley scales of infant development II (BSID-II)	1	Direct assessment	Motor, cognitive and language scales	Yes	Payment required	English	USA
8	Bayley scales of infant development III (BSID-III)	1	Direct assessment	Motor, cognitive and language scales	Yes	Payment required	English	USA
9	Kilifi Developmental Checklist (KDC)	1	Caregiver report	Developmental, locomotor, eye–hand co-ordination, hearing, speech and social–emotional	No	Payment required	Kigiriama	Kenya
10	Lucknow Development Screen for Indian Children	1	Caregiver report	27 milestones: gross motor, fine motor, language and social domains which cover motor, language and social domains which cover each month of age and beyond	Not reported	Not reported	Not reported	Not reported
11	WHO Indicators of Infant and Young Child Development (IYCD)	1	Caregiver report	120 items (23 fine motor, 23 gross motor, 20 receptive language, 24 expressive, language, 30 socioemotional)	Not reported	Not reported	English	Global
12	East Asia-Pacific Early Child Development Scales (EAP-ECDS)	2	Direct assessment	Cognitive development; socio-emotional development; motor development; language and emergent literacy; health, hygiene and safety; cultural knowledge and participation; and approaches to learning	Yes	Free to use	Local language	UNICEF
13	International Development and Early Learning Assessment (IDELA)	2	Direct assessment	Language/literacy, numeracy/cognitive development, physical development and social-emotional development	No	Free to use	English	Save the children
14	Early Childhood Development Index (UNICEF)	2	Caregiver report	(1) language/cognitive (2) physical, (3) socio-emotional and (4) approaches to learning	No	Free to use	English	UNICEF
15	Child Developmental Scale of China (CDSC)	2	Direct assessment	Language, Early Math, Social Cognition and Physical Movement	No	Not reported	Chinese	China
16	Hong Kong early child development scale (HKECDS)	2	Direct assessment	Personal, social and self-care; language development; pre-academic learning; cognitive development; gross motor; fine motor; physical fitness, health and safety; and self and society	No	Not reported	Chinese	China (Hong Kong)
17	Early development instrument (EDI)	2	Direct assessment	Five domains: physical health and well-being, social competence, emotional maturity, language and cognitive development and communication skills and general knowledge	No	Not reported	English	Telethon kids institute Australia
18	Wechsler intelligence scales for children (WISC)	2	Direct assessment	Intelligence (receptive vocabulary, block design, information, object assembly and picture naming)	No	Payment required	English	English
19	Wechsler preschool and primary scale of intelligence-III (WPPSI-III)	2	direct assessment	Intelligence (receptive vocabulary, block design, information, object assembly and picture naming)	No	Payment required	English	USA
20	Measuring early learning quality and outcomes (MELQO) (MODEL module)	2	direct assessment and teacher report	(1) Executive function; (2) Social–emotional development and (3) Pre-academic skills (literacy and mathematics)	No	Free to use	English	Global
21	Peabody picture vocabulary test (PPVT)	2	Direct assessment	Language/literacy; receptive and expressive vocabulary	Yes	Payment required	English	South Africa
22	MacArthur-Bates communicative development inventory	2	Caregiver report	Language. Subscales oral language: vocabulary comprehension, production, gesture use and early grammar	No	Payment required	English	Brookes Publishing
23	McCarthy scales for children’s abilities general cognitive index (MSCA)	2	Caregiver report	General cognitive index (CGI): verbal scale (perceptual-performance, quantitative), memory scale and motor scale	No	Payment required	English	USA
24	British ability scales (BAS)	2	Direct assessment	Cognitive ability and educational achievement	Yes	Payment required	English	UK
25	Sandford Binet	2	Direct assessment	Fluid reasoning, knowledge, quantitative reasoning, visual-spatial processing and working memory	Yes	Payment required	English	France
26	Kaufman assessment battery for children (KABC)	2	Direct assessment	Achievement, simultaneous processing and sequential processing	Not reported	Not reported	English	Not reported
27	Kaufman assessment battery for children – second edition (KABC-II)	2	Direct assessment	Individual’s strengths and weaknesses in cognitive ability and mental processing	Yes	Payment required	English	Not reported
28	Baroda Development Screening Test (BDST)	2	direct assessment	motor and mental development of infants	Yes	Payment required	English	not reported
29	Developmental neuropsychological assessment (NEPSY-II)	2	Direct assessment	Measure of executive functions, memory, language and reasoning	Not reported	not reported	English	Not reported
30	Malawi developmental assessment tool (MDAT)	3	Direct assessment	Gross motor, fine motor, social development and language	Yes	Free to use	English	Malawi and England
31	Ages and stages questionnaire 3rd ed. (ASQ-3)	3	Caregiver report	Cognitive and socio-emotional	No	Payment required	English	Brookes Publishing
32	ICMR (Indian Council Medical Research) Psychosocial Developmental Screening Test	3	Direct assessment	Gross motor, vision and fine motor, hearing, language and concept development and personal	No	Not reported	Not reported	Indian Council of Medical Research
33	Screening Test Battery for Assessment of Psychosocial Development (STBAPD)	2	Direct assessment	5 areas: (1) gross motor, (2) vision and fine motor, (3) hearing language and concept development, (4) self-help skills and (5) social skills	Not reported	Not reported	Not reported	Not reported
34	TDSC (Trivandrum developmental screening chart)	3	Direct assessment	(1) Motor, cognitive and language and (2) gross motor, fine motor-adaptive, personal social and language domains of development	Not reported	Not reported	Not reported	India

**Table 5.** Environmental (Home and ECE centre) assessment tools by name, target age, administration method, domains, battery, accessibility, language and country/Institution.

N	Tool’s name	Tool’s target age	Administration method	Domains	Battery	Accessibility	Language of the tool	Country/Institution origin
Home environment								
1	Infant/toddler (IT) home, early childhood (EC) HOME	3	Direct assessment	Quality of parenting and the home environment	No	Payment required	English	USA
2	Family Care Indicator (FCI) questionnaire	3	Caregiver report	Household books, magazines, varieties and sources of play materials, and play activities	No	Not reported	English	UNICEF
3	Home screening questionnaire (HSQ)	3	Caregiver report	30 items; multiple choice, fill in the blanks, yes/no questions plus a toy inventory checklist. Domains not reported	No	Payment required	English	India
4	Multiple indicator cluster survey (MICS)	3	Caregiver report	Two questionnaires: Household Questionnaire; Questionnaire for Individual Women. Domains not reported	No	Not reported	English	UNICEF
5	Multiple indicator cluster survey (MICS 3)	3	Caregiver report	Cognitive caregiving: reading books, telling stories, naming, counting and drawing; socioemotional caregiving: playing with the child, singing songs and taking the child outside	No	Not reported	Not reported	UNICEF
ECE setting								
1	Infant/toddler environment rating scale-revised (ITERS-R); early childhood environment rating scale-revised (ECERS-R)	3	Direct assessment	ITERS-R: 39 items across seven dimensions: space and furnishings, personal care routines, listening and talking, activities, interaction, program structure and parents and staff; ECERS-R: 43 items across seven subscales: space and furnishings, personal care routines; language-reasoning; activities, interaction, program structure and parents and staff	No	Payment required	English, but has many translations	USA
2	Child activities (CA) system	2	Not reported	Not reported in SLR (Fernand et al., 2009)	Not reported	Not reported	Not reported	World Bank
3	Chinese early childhood environment rating scale (CECERS)	2	Direct assessment	Space and furnishings; personal care routings; curriculum planning and implementation; whole-group instruction; activities; language-reasoning; guidance and interactions; and parents and staff	No	Not reported	Chinese	China
4	Measuring early learning quality and outcomes (MELQO) (MELE module)	2	Direct assessment and teacher report	(1) Environment and physical setting, (2) family and community engagement, (3) personnel; (4) interactions, (5) inclusiveness, (6) pedagogy and (7) play	No	Free to use	English	Global

Overall, our SR highlights a need for improvement in the way studies report a tool’s psychometric properties and the cultural adaptation. In line with McCoy et al. (2018b) we found few valid and reliable tools suitable for use in comparative studies across LMICs for cognition and the environment.

Finally, conducting the SR has raised important questions about how measures are selected. Reliability and validity are necessary dimensions in deciding appropriate measures but equally important are considerations of cultural appropriateness and suitability of the tool for intended use. Making an informed choice about which measure and why requires a nuanced understanding of the purpose and overarching objectives of the project and research focus. Why, what and how to measure children’s development at different ages are crucial decisions to choose suitable ECD measures (Fernald et al., 2009). Our SR has served as a foundation for identifying relevant opportunities and challenges when choosing ECD measures in LMICs. Over 30 years of child development, research has emphasised the ways in which children and contexts shape each other (Sameroff and MacKenzie, 2003) yet studies in LMICs have often been limited to child level measures alone. Any attempt to measure and model development must include both the child and the different contexts in which they develop. The SR confirmed that to capture the child’s biodevelopmental niches measures at child and environment level are needed.

## Limitations

As all SR, the results were determined by our keywords and search parameters. The focus during the last decade meant that resources published before 2010 were excluded. Although we compensated for this focus on the last decade by including nine previous relevant SR, there are limitations derived from our choices. In addition the significant number of studies that did not report their psychometric properties or cultural adaptations limited our ability to synthesise the evidence from these sources.

## Conclusion

Effective ECD measures are crucial for meeting the SDGs. Our SR illustrates a number of opportunities and challenges when identifying tools to measure ECD across LMICs. Selecting appropriate measures is a crucial step to tracking early development and learning to better understand a complex challenge such as childhood stunting. A poorly chosen measure can significantly compromise the best research design and study. Overall our SR put forwards 12 key considerations used to compare the tools. Five dimensions present in Tables 2 and 3 (study, population tested, validity, reliability and cultural adaptability/translation) bring attention to previous applications of the tools in LMICs. Seven dimensions outlined in Tables 4 and 5 (target age, administration method, domains, battery, accessibility, language and country/Institution) refer to the tools’ characteristics. Together they can illuminate the process of selecting assessment tools. These key considerations extend beyond evaluating basic psychometric properties to consider the wider social context in which children are developing to ensure their suitability and validity for the study’s purpose.

Finally, our contribution to the field of early childhood research is the revision of 43 up-to-date tools (34 for assessing children’s development, five to measure the home environment and four to measure ECE settings) for measuring ECD across LMICs. We suggest that the 12 key considerations used in our SR are critical as they offer future researchers and practitioners in the field a guide to pay attention to the implementation challenges, psychometric properties and cultural appropriateness of different tools to assess ECD in LMICs.

## Supplemental Material

sj-pdf-1-ecr-10.1177_1476718X211020031 – Supplemental material for Measuring early child development across low and middle-income countries: A systematic review Supplemental material, sj-pdf-1-ecr-10.1177_1476718X211020031 for Measuring early child development across low and middle-income countries: A systematic review by Bernardita Munoz-Chereau, Lynn Ang, Julie Dockrell, Laura Outhwaite and Claire Heffernan in Journal of Early Childhood Research
