# Supplementary material for: Measuring early child development across low and middle-income countries: A systematic review
Source: J Early Child Res. 2021 Jun 14;19(4):443–70. doi: 10.1177/1476718X211020031 (PMC12160078; doi:10.1177/1476718X211020031)
Supplement: sj-pdf-1-ecr-10.1177_1476718X211020031 – Supplemental material for Measuring early child development across low and middle-income countries: A systematic review [file sj-pdf-1-ecr-10.1177_1476718X211020031.pdf]

Appendix 1: Tool information, studies and tool application coded in the qualitative synthesis

**Tool information**	
Domain	Development early childhood assessment tools that measure (1) child's development or (2) learning environment
Content	Description of the scales included in the tool (i.e language, cognitive development)
Name	Name of the early childhood assessment tool. When two tools have been developed by the same team and complement covering different age ranges, revise these together
Target age	Ages that the tool can be applied according to the tool makers
Completion	Who should complete the tool? (i.e trained enumerator, caregiver report, parents/ECD practitioners)
Battery	Does the tool include a battery needed for its application? (Yes/No)
Accessibility	Is the tool free to use, or a payment (i.e copyright) needs to be done? (Yes/No)
Language	Language in which the tool was originally created
Country/Institution	Country/Institution where the tool was created
**Study information**	
Year	Year in which the article was published
Authors	Name of the authors of the article
Institutions	Institutions (i.e Universities, research Centres) where authors are affiliated
Title	Journal article's title
**Tool application**	
Population tested	Country/Region where the tool was implemented
Sample size	N of participants (i.e number of children, parents/caregivers, centres)
Age and range	Age of the children measured by the tools used in the study
Validity	Psychometric properties concerning the tools' validity
Reliability	Psychometric properties concerning the tools' reliability (i.e Test–retest; Inter-rater; Internal (inter-item correlations or Cronbach's alpha)
Cultural adaptation	Description of the linguistic equivalence (translation) and/or cultural equivalence (consideration of the local cultural environment)
Results and conclusions	Main results reached by the authors concerning the tools applied
Purpose	Domains to be assessed by the tool in the study
Setting	Where was the tool administered? (i.e home, ECCE centre, clinic)
Time	Length/time for administration of the tool in the study
Training	Description of the training provided by the study
Quality appraisal	Quality of the tool provided by the authors of the study
